# Improving the Drilling Parameter Optimization Method Based on the Fireworks Algorithm

**DOI:** 10.1021/acsomega.2c05692

**Published:** 2022-10-14

**Authors:** Li Yang, Zhuohui Lu, Weijian Ren, Tianyi Liu

**Affiliations:** College of Electrical Information Engineering, Northeast Petroleum University, Daqing163318, China

## Abstract

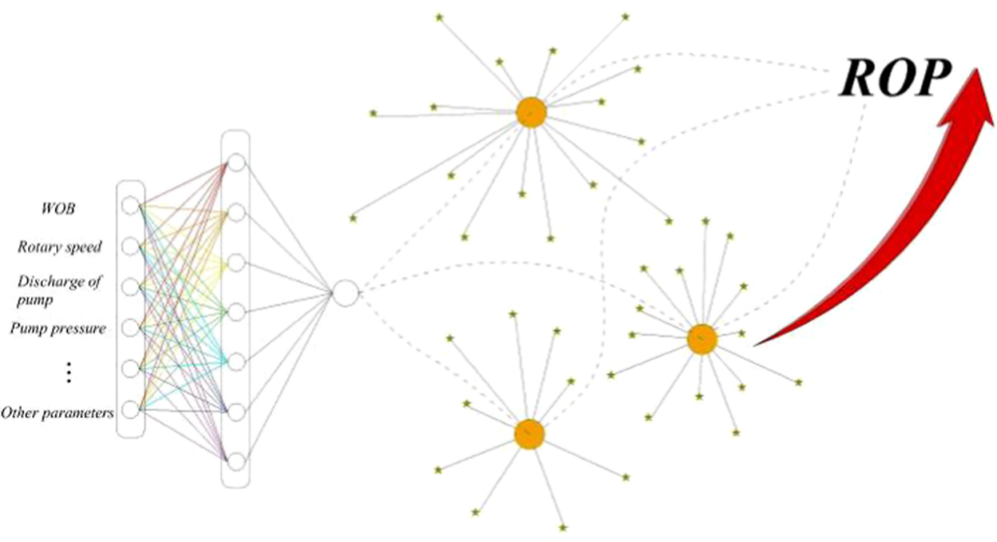

The rate of penetration (ROP) is a manifestation of drilling efficiency, and optimizing drilling parameters is an important way to improve it. To achieve a low ROP for a Permian formation in a certain oil and gas field, three single wells in this formation were selected for optimization. An improved fireworks optimization algorithm was proposed for drilling parameter optimization. We first established the objective function that predicted the ROPs for the three wells. The objective function employed a multilayer perceptron neural network as the optimization adaptation function. We then optimized four controllable parameters (weight on bit, rotary speed, pump discharge, and pump pressure) and improved the fireworks algorithm with an adaptive number of various factors. This improvement enhanced the debugging performance of the fireworks algorithm during optimization. The results indicated that the improved fireworks algorithm has significantly enhanced search performance, and the optimum ROPs for the three wells were increased by 38.55, 78.30, and 60.15%, which provides a reference for the controllable parameter setting in the area.

## I Introduction

Improving drilling efficiency has always been one of the most effective ways of reducing drilling costs. Researchers have intensely pursued improving drilling technology, and the most critical factor affecting drilling efficiency is the rate of penetration (ROP). ROP optimization is broadly divided into two parts: (1) optimizing the structural combination of oil and gas wells according to the specific geological environment, including stratigraphy and rock, and (2) optimizing controllable drilling parameters.^1^ For example, Xing^2^ analyzed and optimized ROP improvement and coring efficiency technologies, and Zhang et al.^3^ compared and selected different combinations of drilling tools to improve the ROP. Meanwhile, Ravela et al.^4^ considered that excessive ROP might cause complex situations and increase production time while optimizing ROP; hence, they proposed a hybrid data-driven model to optimize both ROP maximization and nonproduction time minimization. Wang^5^ analyzed the optimal ranges of drilling parameters for different drill pipe stresses. Mustafa et al.^6^ used the response surface method to develop a mathematical model of the controllable drilling parameters and determine their optimal ranges. Darwesh et al.^7^ used multiple linear regression model training to obtain the optimal weightings of bit, rotary speed, and ROP. Hegde et al.^8^ considered the effect of drilling vibration in optimizing drilling parameters while mitigating excessive vibrations.

The progress of intelligent technology has transformed optimization from mathematical models to intelligent algorithm optimization. Population algorithm optimization has become mainstream among algorithmic optimization techniques.^9^ This optimization technique is generally inspired by imitating the optimization method of plants and animals and natural phenomena for different effects. Each optimization method has a unique optimization strategy, which can reduce the convergence time during programming calculations, and they can be employed for optimizing controllable drilling parameters. Many scholars have built on these strategies with old or new and more suitable optimization algorithms. For example, Zheng et al.^10^ added the cellular automata mechanism to the particle swarm algorithm to optimize the three objectives of ROP, mechanical specific energy, and bit life simultaneously, and Jing et al.^11^ added a pattern search process led by a genetic algorithm to improve the ability to local optima. Moazzeni et al.^12^ used a hybrid bat algorithm to prevent the algorithm from falling into a local optimum to a certain extent. Although the above studies have innovative optimization methods, they use traditional ROP equations in establishing the objective function of the optimization object, which is very dependent on the parameter settings given by experts. In contrast, ROP prediction models with big data analysis are independent of parameter settings. For example, Gan et al.^13^ used machine learning methods for ROP prediction and optimized the ROP by the rainwater optimization algorithm using the wide range of raindrops in a global optimization search with good results. This study applies the fireworks optimization algorithm to the optimization of drilling parameters. This technique optimizes with sparks generated by firework explosions. The technique is simple and efficient, has parallel distribution, does not easily fall into local optima, and has achieved good optimization results in this study.

There are four issues to be considered when optimizing drilling parameters: (1) the relationship between parameters and objectives, (2) the setting of constraints, (3) the feasibility of the proposed optimization method, and (4) instance verification. The relationship between parameters and objectives is the most direct way to evaluate the optimization process and the optimized index; the constraints constrain the range of parameters values to avoid complications and errors caused by too large or too small parameters; finally, the optimization method is key to solving the optimization problem, but most optimization algorithms cannot be directly applied to a particular area and must be combined with the specific modeling problems to be improved.

The manuscript is structured as follows. We focus on establishing the fitness function in Section II. The optimization algorithm and its improvement is then presented in Section III. The experiments with examples are given in Section IV. Finally, the study is summarized in Section V.

## II Adaptation Function Setting Based on the MLP Neural Network

Regression problems are suitable for finding the relationship between input and output quantities, especially when the input value changes to predict the exact output. Similarly, in optimization problems, a suitable prediction model is essential to get the best output accurately. The fitness function is an important indicator of ROP optimization, and by the same token, it is also the result of judging the specific quantification of the optimized parameters. Previously, the ROP equation^14^ and mechanical specific energy^15−17^ have been the main methods to establish the optimization index (i.e., the fitness function), but because of their excessive dependence on formation information, they require engineers to give the corresponding parameter settings through experiments, which brings considerable difficulty to optimize the ROP. With the popularization of big data analysis methods, ROP prediction models based on machine learning and deep learning have gradually become new research techniques, which calculate and generalize the laws existing in the data to reach the predicted ROP values under similar conditions by simply feeding the controllable parameters into the trained model.^18^ For example, Ahmed et al.^19^ used an artificial neural network (ANN) to predict ROP. Kor et al.^20^ compared different prediction methods based on a statistics viewpoint and proved the effectiveness of support vector machine regression in ROP prediction. Ashrafi et al.^21^ obtained the weights of each neuron connection of the ANN through an optimization algorithm, thus replacing the backpropagation algorithm. The results indicated that the effect of particle swarm optimization on the multilayer perceptron (MLP) network is superior. Compared with the ROP equation as the objective function of ROP optimization, the ROP prediction model obtained by big data analysis and processing is simpler and more accurate.

### II.I Data Analysis

The data set was collected from the surface to several kilometers underground, including each well’s completion reports, history, detailed records of the equipment models, parameter configuration, accidents, and other related information. The data were acquired by obtaining the average value of all drilling parameters each time there was drilling, from one stratum to another, or when the bits were replaced. Owing to the complexity of the formation, the same drilling conditions could occur in adjacent formations. Therefore, some data indicate continuous drilling in two or more formations. The average parameters of each drill were also used to analyze the data. In this paper, because the data is confidential, all words and names related to sensitive drilling data are blurred, and only the application results of the study are presented.

In this study, vertical well section data were collected from 21 wells drilled in one oil and gas field. The database consists of 1015 data points, and the data includes parameters such as weight on bit (WOB). The controllable parameters used in this study include WOB, rotary speed, pump discharge, and pump pressure; uncontrollable parameters mainly include drilling time, starting depth, ending depth, and drilling diameter. These parameters are regarded as input vectors in the related model, and the output-dependent variable defaults to ROP.

Table 1 presents a list of statistics for the eight independent parameters and the dependent variable (i.e., ROP). Variation range, mean, and deviation are some of the characteristics shown in the table.

**Table 1:** Statistics of the Data Set

parameter	min	max	average	deviation
WOB (kN)	3.00	300.00	72.08	58.39
rotary speed (rpm)	0.36	130.00	51.57	20.01
pump discharge (L/s)	8.40	75.00	28.07	16.01
pump pressure (MPa)	1.00	27.00	17.63	4.64
starting depth (m)	0.00	8326.00	5550.82	2190.86
ending depth (m)	53.35	8433.00	5887.69	1903.42
drilling time (h)	0.50	6480.00	775.22	1205.64
drilling diameter (mm)	118.00	660.40	226.81	87.31
ROP (m/h)	0.12	157.50	9.29	20.14

**Figure 1:**
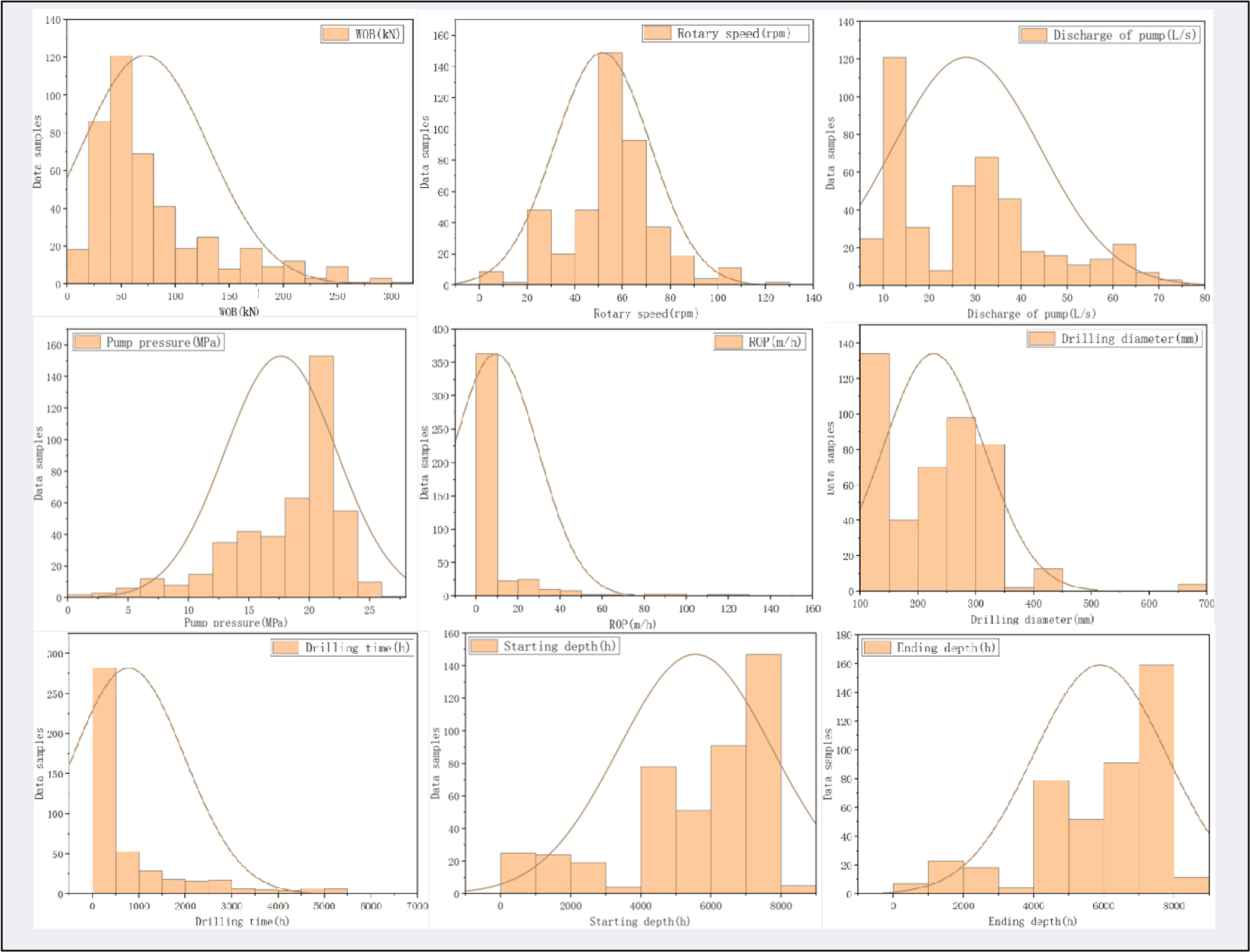
Distribution of the complete data set utilized for model development.

Figure 1 shows the distribution of various parameters at different intervals. Notably, in the statistical process, owing to the influence of deep wells and ultradeep wells, most of the ROP values are low level. Among the controllable parameters, the WOB is common when drilling at low pressures.

### II.II MLP Neural Network Prediction Model

Multilayer perceptron (MLP) neural networks, also known as ANNs, are one of the most effective and popular models in dealing with text-based big data fitting or classification problems. They are also one of the most widely used models for machine learning applications in ROP prediction. They consist of input, hidden, and output layers. The layers are connected by neurons, and weights and biases are used to ensure the transmission of information. The weights and biases are trained by backpropagation to give the model judgment and prediction abilities.

**Figure 2:**
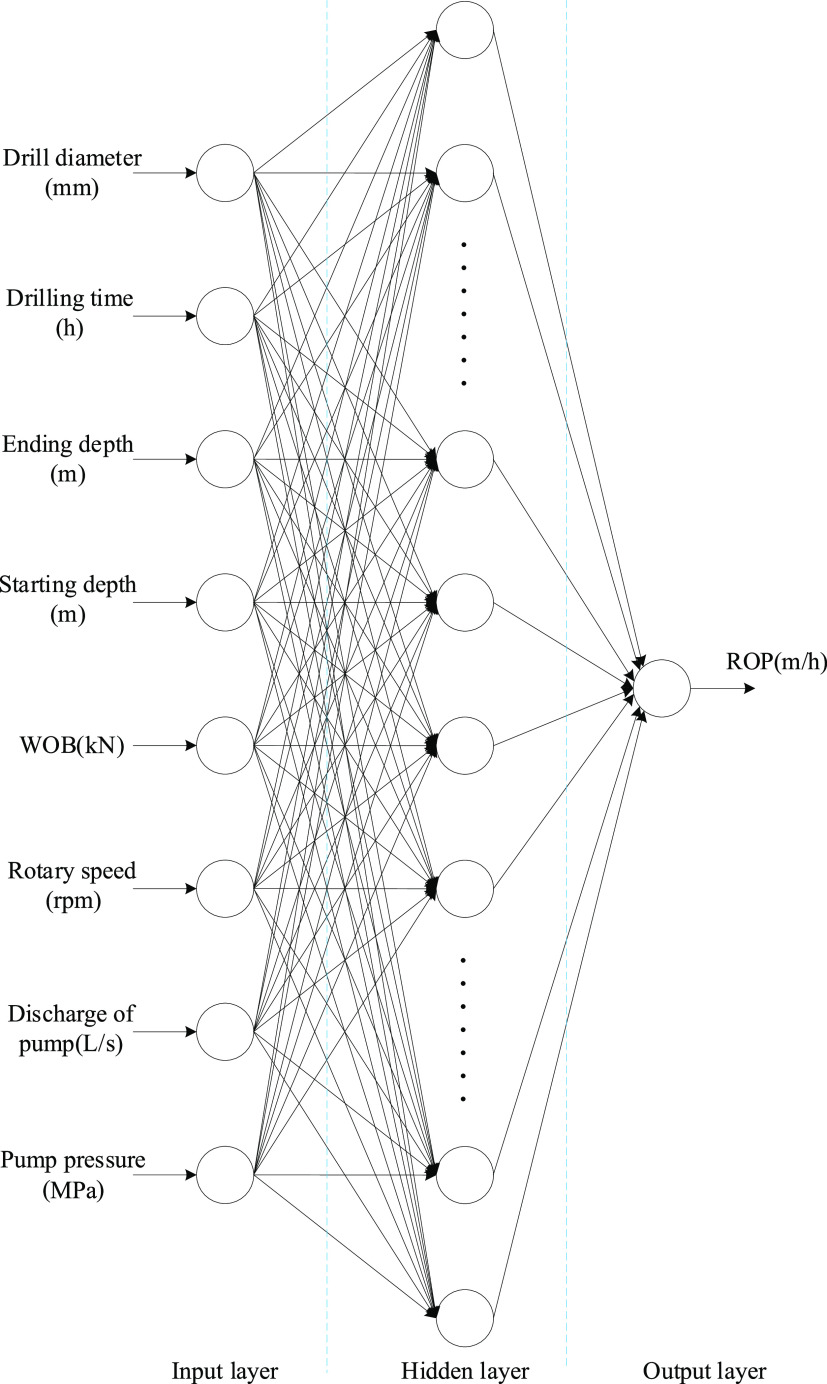
Drill speed prediction model applying the multilayer perceptron (MLP) model.

Controllable factors and a variety of other factors must be considered and combined for training. The data used for training and testing in this study are from multiple straight wells in a well area in the Xinjiang region. The training parameters fed into the model for the input layer are the drill diameter, ending depth, starting depth, drilling time, and controllable parameters, and the output layer is ROP. The numbers of neurons in the input, hidden, and output layers are 8, 20, and 1, respectively, and the structure of the model is shown in Figure 2.

### II.III Predictive Model Evaluation

The Scikit-learn package is a general-purpose, open-source machine learning library that covers almost all machine learning algorithms and can be used to build an efficient data mining framework. It provides convenient and rigorous multilayer perceptron prediction models as a reference to facilitate model building. First, the range between parameters in the original data does not belong to the same order of magnitude, and all feature sizes must be converted to similar intervals to eliminate the effect of magnitude. sklearn.preprocessing provides three common data preprocessing methods, and several experiments have verified that its superiority of fit performance according to StandardScaler is 0.96 and 0.59 and −0.13 according to Normalizer and MinMaxScaler, respectively, where the StandardScalar method has the best mean square error (MSE) and mean absolute error (MAE). Next, the data set is divided into training and validation sets, and the training set is divided into ten copies. The training and testing sets are divided according to the 9:1 ratio ten times, and the testing set is one of the nine training sets for each iteration. The old testing set is automatically put into the new training set for tenfold cross validation; finally, logistic is chosen as the activation function in the parameter setting, and the optimizer is chosen. The loss function is the squared loss function; the training method is the mini-batch gradient descent method, wherein the batch size =10. Here, we chose the adam optimizer with a learning rate of 0.01 and a stop training error of 10^–3^.

The model was evaluated using MSE, mean absolute error MAE, and goodness-of-fit *R*^2^. Table 2 lists the detailed results of the ten experiments.

**Table 2:** Predictive Model Evaluation Results

evaluation metric	expt 1	expt 2	expt 3	expt 4	expt 5	expt 6	expt 7	expt 8	expt 9	expt 10
MAE	0.88	1.54	1.79	0.74	5.63	3.08	0.93	5.65	0.67	0.88
MSE	0.56	0.92	0.69	0.55	0.91	0.58	0.56	0.85	0.49	0.48
*R*^2^	0.94	0.91	0.81	0.96	0.96	0.85	0.95	0.73	0.96	0.95

Most of the experimental results have good prediction ability, except for a few iterations with average prediction capabilities (e.g., groups 3, 6, and 8). Among the experiments, group 9 has the best experimental results and is chosen as the fitness function of the following optimization model.

**Figure 3:**
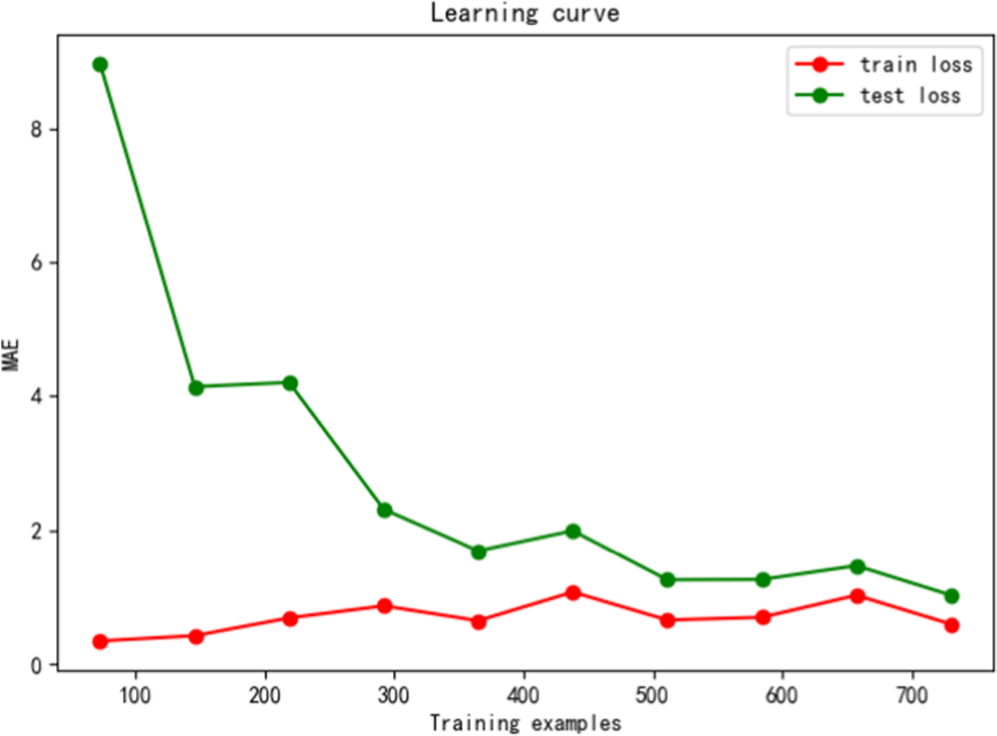
MLP model learning curve.

The fitting effect of the model can be verified using these two aspects: the learning curve and the results of the validation set. First, we used the training and testing set errors to define our curve. As shown in Figure 3, the average absolute errors of the training and testing sets are very low.

**Figure 4:**
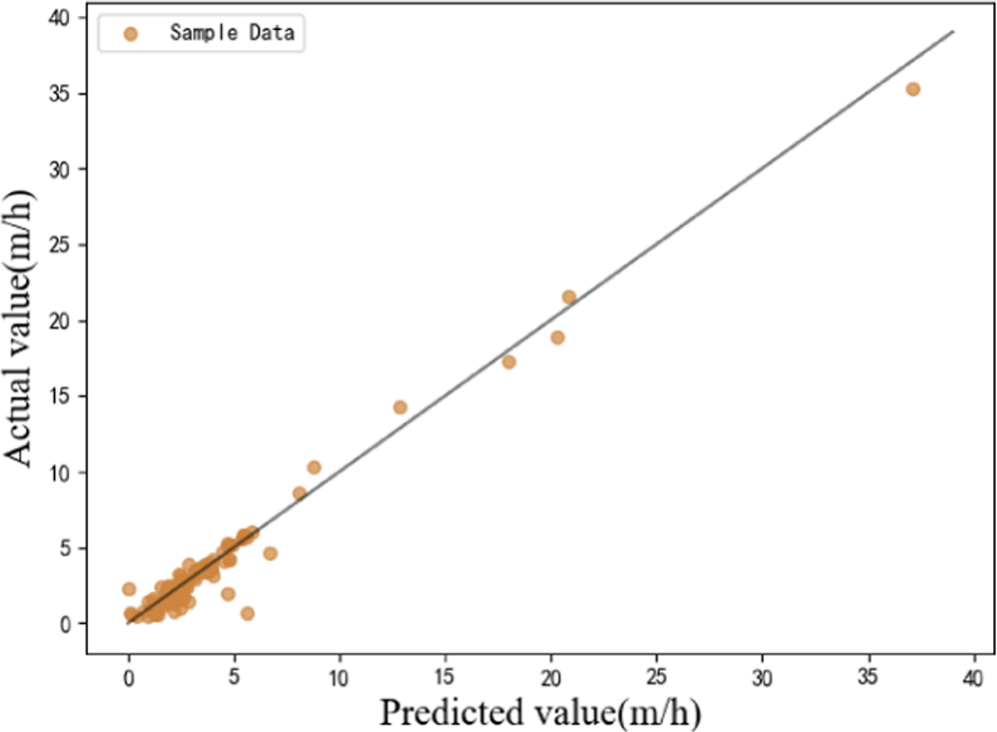
Relationship between actual and predicted values.

Second, the model fitting condition was evaluated by the verification set fitting effect. The validation set data is not included in model training; thus, the validation set results can be used as the indicator for evaluating the model. Figure 4 shows the relationship between the actual and predicted values of the experimental validation set. The abscissa and ordinate of Figure 4 represent the predicted and actual values, respectively. When the data points are closer to the oblique 45° straight line, the fitting effect of the model is better. The performance metrics *R*^2^, MAE, and MSE for the validation set were 0.96, 0.54, and 0.73, respectively. The results indicated that the MLP model has excellent prediction ability.

## III Introduction and Improvement of the Fireworks Algorithm

### III.I Fireworks Algorithm

The fireworks algorithm was proposed by Professor Tan of Peking University in 2010.^22^ It is an optimization algorithm created by imitating fireworks explosions to generate sparks, which demonstrates its unique search mechanism and reduces the search time compared to other optimization algorithms. The algorithm involves three operations: explosion, variation, and selection,^23^ where the explosion process can be seen as a process of searching in the local space around the explosion using the sparks generated. During the variation operation, each firework explodes to generate several sparks, and certain sparks have a probability of generating a special spark that increases population diversity. The selection operation searches among the fireworks, exploding sparks, and variation sparks. Its selects a number of sparks as the initial sparklers for the next iteration, where the most adapted individuals are directly used as sparklers for the next iteration. The remaining sparklers are selected probabilistically using roulettes. Sparks are randomly and uniformly distributed around that firework according to the amplitude of the current spark, while the variant sparks are generated in a normally distributed manner around the current spark.

Each sparkle in the fireworks algorithm has two properties: the amplitude *A*_*i*_ and the number of explosions *s*_*i*_. The brighter the sparkle (the better the target), the smaller the amplitude; simultaneously, the brighter the sparkle produced by the explosion (the better the target), the greater the number of sparks produced by allowing it to explode.

The formula for calculating the number of sparks is
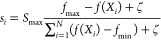
1
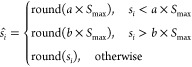
2where *S*_max_ is the maximum number of sparks generated by the explosion, *f*(*X_i_*) is the current value of the adaptability of the fireworks, *f*_max_ and *f*_min_ are the maximum and minimum values of the adaptability of the firework population, respectively, *a* is the minimum spark generation ratio, *b* is the maximum spark generation ratio, ξ is a very small real number that prevents the denominator from being 0, and the round() function is a rounding function based on the rounding principle.

The amplitude is calculated using the formula
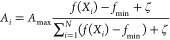
3where *A*_*i*_ represents the amplitude of the *i*th firework and *A*_max_ is the maximum amplitude. The location of the sparks produced by each firework is
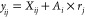
4where *r* is a random in the range of [−1, 1].

Each iteration generates *m* special fireworks; that is, *m* fireworks are randomly selected among *N* fireworks, and a special spark is generated by mutating each spark corresponding to the selected fireworks. The special spark is generated by eq 5
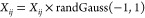
5where randGauss(−1, 1) is a Gaussian-distributed random number with a mean and variance of 1.

### III.II Improved Fireworks Algorithm

The fireworks algorithm produced good experimental results, but the number of explosions was almost always small; that is, most of the fireworks in the process of finding the best probability cannot effectively search for a better solution, and in the next iteration of the fireworks, the best individual from the previous generation and a roulette individual are selected, reducing the probability of contacting the optimal in the next generation of firework explosions. Hence, the fireworks algorithm’s optimization performance must be improved.

To effectively solve the search for the optimal solution with fewer fireworks, we proposed an adaptive variation factor fireworks algorithm (AVFFWA). This algorithm sets a reference value for the number of explosions, and if the number of explosions for all fireworks in an iteration is less than the reference value, the number of sparks generated by the variation is increased by 1, and for the opposite case, the number of variation sparks is not changed. The specific steps to improving the fireworks algorithm are as follows:

(1) initialize the number and location of fireworks, number of exploding sparks, upper and lower limits of the number of constrained sparks, radius of the explosion, and number of variant sparks;
(2) generate exploding and Gaussian variant sparks;
(3) determine whether the number of sparks generated by the fireworks is greater than the set parameter value; if it is less than the parameter value, the number of variable sparks generated is increased by one; and for the opposite case, the number of variable sparks is not changed;
(4) calculate the fitness function of fireworks, exploding sparks, and variant sparks, and use the optimal individual and some of the other individuals as the initial fireworks for the next iteration; determine whether the fitness value of the optimal individual is greater than the historical optimal value; if it is, the historical optimal value is updated, and retained for the opposite case; and
(5) evaluate whether the end condition is satisfied; if it is satisfied, output the optimal position and value; if not, return (2).

**Figure 5:**
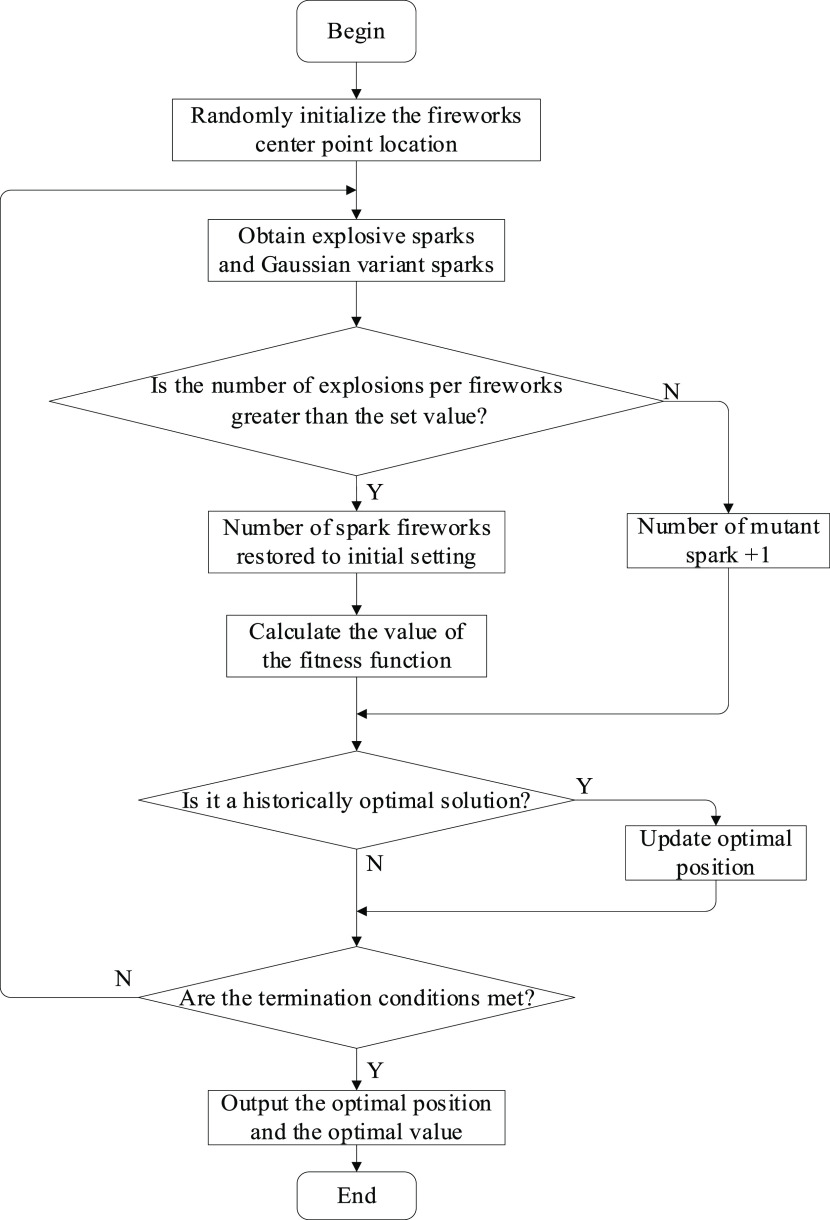
Flow chart of the improved fireworks algorithm.

The specific flow chart is shown in Figure 5.

### III.III Drilling Parameter Optimization

To illustrate the feasibility of the design method, we selected controllable parameters in a well area of a certain oil and gas field in the Permian formation for optimization and established a drilling parameter optimization model based on the improved fireworks algorithm. We also performed simulation experiments. The experiment fixes the uncontrollable parameters of the prediction model (ending depth, starting depth, drill diameter, and drilling time) and finds the optimal combination of parameters and ROP values under the constraints by changing the drilling parameters (weight on bit, rotary speed, pump discharge, and pump pressure).

### III.IV Design Well Overview

Three adjacent wells were optimized for this experiment. The wells had the formation lithology of gray, gray, and gray-green tuffs in the upper part and gray-green inclusions; black, gray-green, brown, and brown-red (crystalline and glassy) tuffs; and tuffaceous mudstones and sandstones in the middle and lower parts with drilling depths from 120 to 220 m. All drill bits used were tooth wheel bits. After a comparative analysis of the ROP values of other data in the area, the ROP of three designed wells was low, and optimization was feasible.

The detailed parameters of the design wells are listed in Table 3.

**Table 3:** Design Well Parameters

design well no.	drill diameter (mm)	drilling time (h)	starting depth (m)	ending depth (m)	WOB (kN)	rotary speed (rpm)	pump discharge (L/s)	pump pressure (MPa)	ROP (m/h)
1	311.2	125.5	4589	4813.84	280	60	50	20	1.79
2	311.2	38.53	4435.08	4557.79	250	60	40	15	3.18
3	250.8	87	4580.03	4806.75	20	60	32	22	2.61

The drilling data cover almost all of the parameters taken in the normal working condition, so the maximum and minimum values of the parameters in the data are used as the constraint range for the optimization. In addition, the parameters outside the range do not participate in the neural network training, and the prediction accuracy is unknown for this part of the model and is not involved in learning. The parameters within the constraint range can satisfy the conditions of the optimization search.

### III.V Optimization of Drilling Parameters Based on the Improved Fireworks Algorithm

Each design well needs to be standardized before being optimized with the model, and the corresponding well data is divided into the first four uncontrollable parameters and the last four controllable parameters. The first four uncontrollable parameters of the design well are fixed by programming, and the fireworks, sparks, and variable spark locations generated by each iteration are added to the uncontrollable parameters after the standardization process to form one or more complete datasets to be predicted by the prediction model for prediction.

The parameters of the fireworks algorithm are set as follows: the initial number of fireworks is 10, the fireworks dimension is 4, the number of explosions is 50, upper and lower limit coefficients for the number of explosions are 0.4 and 2, respectively, the maximum amplitude is 4, the number of variable sparks is 5, and the number of iterations is 1000.

**Figure 6:**
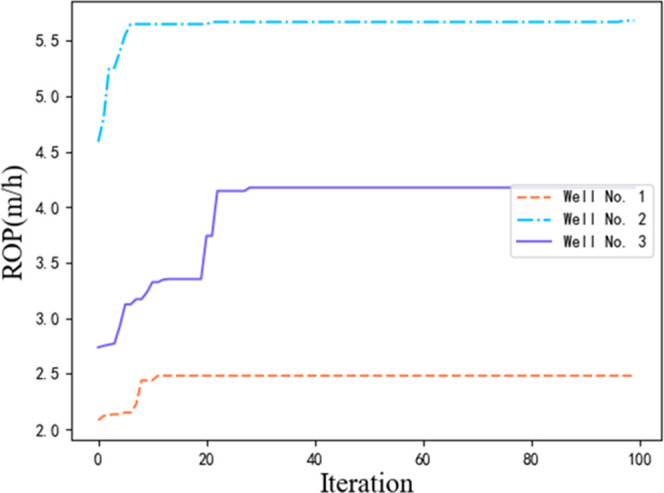
Improving the fireworks algorithm optimization process.

The improved fireworks algorithm explosion has 30 detection values, and if the number of generated explosive sparks is less than 30, the number of variable sparks is increased. The optimization process of the three test wells is shown in Figure 6.

Since there is no change in the improved fireworks algorithm after stabilization within the number of iterations, Figure 5 only shows the change in the fitness value for 100 iterations. The experimental results indicated that each test well could find the optimal value quickly after several iterations, as well as the next optimal value.

## IV Results and Discussion

To prove the superiority of the method, we compared the optimization of the improved fireworks, fireworks, and particle swarm algorithms mentioned in ref (8). We designed ten experiments for all methods, and the average and optimal results of each experiment were obtained for analysis and discussion.

**Figure 7:**
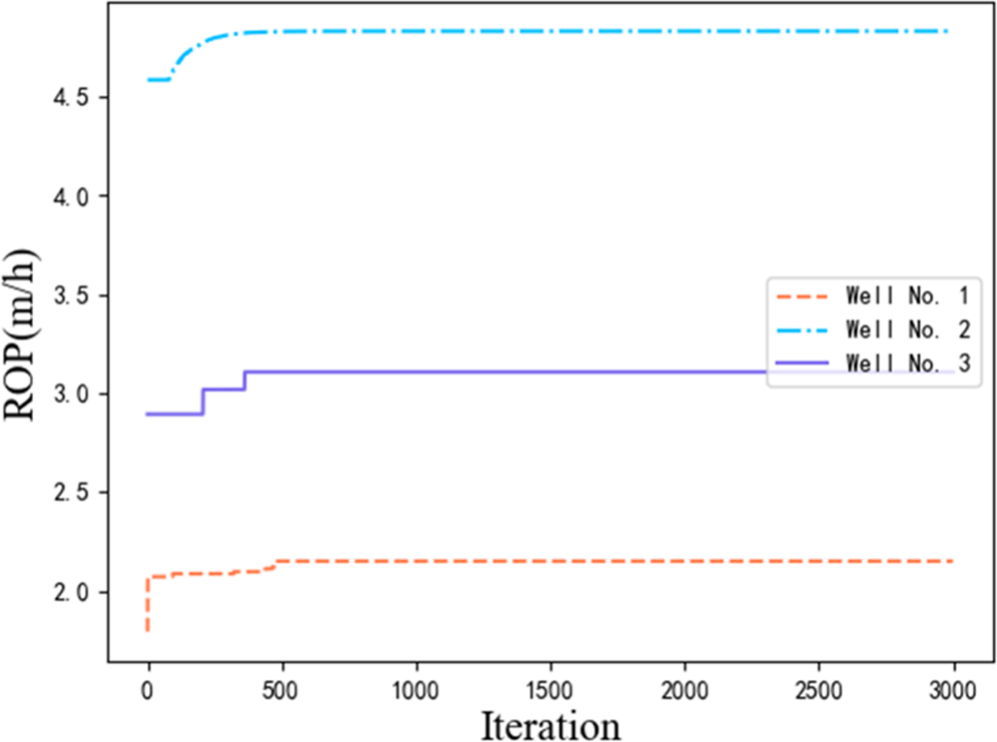
Particle swarm algorithm optimization process.

The parameters of the particle swarm optimization algorithm for wells 1 and 2 were set as follows: individual speed update learning factor of 0.008, global speed update learning factor of 0.008, own speed weight factor set to 0.0016, population size set to 100, and iteration number set to 3000; for well 3, both individual and global speed update learning factors were 0.01, own speed weight factor was 0.05, and the rest of the parameters were the same as in the first and second wells. The optimization process of the particle swarm algorithm is shown in Figure 7.

**Figure 8:**
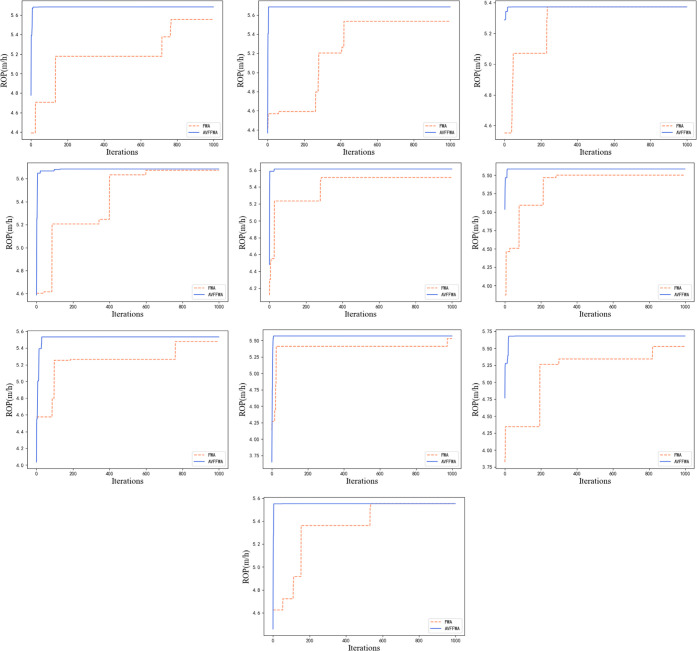
Comparison of the optimization process between the improved fireworks and fireworks algorithms.

Tables 4–6 present the optimal and average ROP results for different optimization methods under ten experiments, which demonstrate the optimization performance of the algorithms and provide detailed parameter values for controllable parameters at the optimal ROP. All three optimization methods converged to long-term stability within the number of iterations.

**Table 4:** Optimization Results of Drilling Parameters of Well No. 1

method	WOB (kN)	rotary speed (rpm)	pump discharge (L/s)	pump pressure (MPa)	actual ROP (m/h)	optimum results (m/h)	average results (m/h)	optimization margin (%)
FWA	285.43	80.00	27.00	22.00	1.79	2.48	2.32	38.55
AFWA	285.41	79.85	32.94	22.00	1.79	2.48	2.48	38.55
PSO	281.56	68.21	30.14	21.95	1.79	2.15	2.11	20.11

**Table 5:** Optimization Results of Drilling Parameters of Well No. 2

method	WOB (kN)	rotary speed (rpm)	pump discharge (L/s)	pump pressure (MPa)	actual ROP (m/h)	optimum results (m/h)	average results (m/h)	optimization margin (%)
FWA	300.85	30.00	35.33	22.86	3.18	5.53	5.44	73.90
AFWA	299.27	31.98	34.92	22.00	3.18	5.67	5.46	78.30
PSO	288.66	55.15	44.68	18.37	3.18	4.93	4.61	55.03

**Table 6:** Optimization Results of Drilling Parameters of Well No. 3

method	WOB (kN)	rotary speed (rpm)	pump discharge (L/s)	pump pressure (MPa)	actual ROP (m/h)	optimum results (m/h)	average results (m/h)	optimization margin (%)
FWA	300.00	30.00	27	22.00	2.61	4.17	4.09	59.77
AFWA	296.11	30.00	28.88	22.00	2.61	4.18	4.18	60.15
PSO	246.87	31.36	36.64	21.62	2.61	3.11	2.98	19.16

The differences in search methods between the fireworks and particle swarm algorithms in dealing with discrete optimization problems cause differences in the optimization results. The particle swarm algorithm easily falls into the local optima in the search, changing the search route. The fireworks algorithm avoids this to a certain extent because of its distributed parallelism and local coverage, which reduce the possibility of falling into a local optimum and enable local search.

A comparison of the improved fireworks algorithm with the original algorithm in terms of search capability is shown in Figure 8.

Figure 8 shows the optimization search process of the fireworks and improved fireworks algorithms under ten well experiments. For the same parameter settings, the fireworks algorithm with a normal optimization search process was slower than the improved fireworks algorithm. Table 7 lists the epoch of the three methods when the ROP changes in ten experiments. The fireworks and improved fireworks algorithms were able to quickly find a better ROP in the optimization process, whereas the particle swarm algorithm generally needed many iterations to continue. The particle swarm algorithm randomly generates particles in the initial space, which has a high probability of directly contacting the better ROP, whereas the fireworks algorithm generates fireworks. The fireworks are used as the center of explosion for optimization, thereby quickly obtaining the next optimal value.

**Table 7:** Epoch of the Three Methods When the ROP Changes

	PSO			FWA			AVFFWA		
trial no.	well no. 1	well no. 2	well no. 3	well no. 1	well no. 2	well no. 3	well no. 1	well no. 2	well no. 3
1	2	237	27	6	2	3	2	2	2
2	2	308	86	8	2	2	2	2	2
3	6	2	95	2	2	2	2	4	2
4	31	324	18	2	2	2	2	2	2
5	20	265	3	2	4	2	2	2	2
6	2	232	77	2	2	2	2	2	2
7	2	2	96	2	2	2	6	2	2
8	25	248	35	2	4	12	2	2	2
9	2	305	72	10	2	2	2	2	2
10	14	2	18	2	2	2	2	2	2

Table 8 lists the epoch of the three methods at the optimal ROP in ten experiments. The average epoch values of the improved fireworks algorithm in three wells are 92.2, 92.6, and 65.1, respectively, which are significantly less than those of the other methods.

**Table 8:** Epoch of the Three Methods When the Optimal ROP Is Reached

	PSO			FWA			AVFFWA		
trial number	well 1	well 2	well 3	well 1	well 2	well 3	well 1	well 2	well 3
1	445	531	298	**445**	793	**114**	113	196	207
2	220	587	396	555	447	432	182	205	27
3	603	506	435	603	**236**	188	116	41	**9**
4	**177**	570	473	560	612	719	12	131	29
5	486	542	355	572	343	409	14	77	19
6	564	492	371	729	351	276	79	**20**	36
7	521	495	**289**	796	785	521	227	71	96
8	513	484	426	833	976	532	9	39	30
9	571	533	297	753	824	614	7	76	35
10	498	**357**	374	810	511	924	163	70	163
average	459.8	509.7	371.4	665.6	587.8	472.9	92.2	92.6	65.1

In general, almost all of the swarm optimization algorithms can be improved by infinitely increasing the number of populations to improve the optimization search capability, but this is undesirable because of computational complexity and invalid search. However, the number of adaptive variant sparks can determine whether to increase the variant sparks according to the current number of fireworks explosions. In the short term, the computational volume and time of this method are slightly greater than the original method, but from the experimental results, the improved fireworks algorithm has a significant improvement in the search performance compared to the original algorithm and can achieve the optimal result with fewer iterations. The improved fireworks algorithm also has a small advantage in terms of the search results.

## V Conclusions

To resolve the problem of low ROP in some wells in the Permian formation of an oil and gas field, this paper proposes a new improved fireworks algorithm (AVFFWA) to optimize the drilling parameters and increase the ROP. The framework of drilling parameter optimization consists of two parts: ROP prediction and drilling parameter optimization. The following conclusions were drawn:

(1) The multilayer perceptron neural network ROP prediction model was used as the fitness function for optimization and predicted by analyzing data. This enables the engineers to be less reliant on the ROP equation by providing specific ground parameter values through actual drilling data.
(2) The fireworks algorithm was first proposed to optimize drilling parameters, and the fireworks algorithm’s ability to search was improved by adaptively varying the number of sparks. The improved fireworks algorithm was compared with the traditional version and the particle swarm algorithm. The results show that the improved fireworks algorithm considerably enhanced the optimization ability for the three test wells.
(3) The optimized ROP can be used as a reference for setting parameters for subsequent drilling operations in similar formations in the region. This method can also provide technical support for parameter optimization in drilling projects elsewhere.

## References

[ref1] Hu Z. J.; Guo M. J.; Lv M. J. Analysis of the development status and trends of multi-objective optimization technology for drilling parameters. China Pet. Chem. Stand. Qual. 2021, 41, 185–188.

[ref2] Xing Z. T. On the application of supporting technology for speeding up drilling of deep bedrock sections in the LT block. West China Explor. Eng. 2022, 34, 47–51.

[ref3] Zhang J. W.; Ji G. D.; Guo W. H.; Wang H. G.; Cui M. Study on Bottom Hole Assembly and Drilling Parameter Optimization for Reaming While Drilling. China Pet. Mach. 2021, 49, 22–27.

[ref4] Ravela S.; Alali A. M.; Abughaban M. F.; Aman B. M. Hybrid Data Driven Drilling and Rate of Penetration Optimization. J. Pet. Sci. Eng. 2020, 200, 108075 10.1016/j.petrol.2020.108075.

[ref5] Wang Y. J. Optimization of Drilling Parameters for Ultra Deep Wells Based on Fatigue Strength Theory. Yunnan Chem. Technol. 2021, 48, 113–115.

[ref6] Mustafa A. B.; Abbas A. K.; Alsaba M.; Alameen M. Improving drilling performance through optimizing controllable drilling parameters. J. Pet. Explor. Prod. Technol. 2021, 11, 1223–1232. 10.1007/s13202-021-01116-2.

[ref7] Darwesh A. K.; Rasmussen T. M.; Al-Ansari N. Controllable drilling parameter optimization for roller cone and polycrystalline diamond bits. J. Pet. Explor. Prod. Technol. 2020, 10, 1657–1674. 10.1007/s13202-019-00823-1.

[ref8] Hegde C.; Millwater H.; Pyrcz M.; Daigle H.; Gray K. Rate of penetration (ROP) optimization in drilling with vibration control. J. Nat. Gas Sci. Eng. 2019, 67, 71–81. 10.1016/j.jngse.2019.04.017.

[ref9] Barbosa L. F. F. M.; Nascimento A.; Mathias M. H.; de Carvalho J. A. Machine learning methods applied to drilling rate of penetration prediction and optimization - A review. J. Pet. Sci. Eng. 2019, 183, 106332 10.1016/j.petrol.2019.106332.

[ref10] Zheng J.; Li Z. L.; Dou B.; Lu C. Multi-objective cellular particle swarm optimization and RBF for drilling parameters optimization. Math. Biosci. Eng. 2019, 16, 1258–1279. 10.3934/mbe.2019061. 30947419

[ref11] Jing N.; Jiang L. M.; Geng Z.; Deng L. C. Drilling Parameter Optimization Based on Advanced Genetic Algorithm. China Pet. Mach. 2016, 44, 10–15.

[ref12] Moazzeni A. R.; Khamehchi E. Rain optimization algorithm (ROA), A new metaheuristic method for drilling optimization solutions. J. Pet. Sci. Eng. 2020, 195, 107512 10.1016/j.petrol.2020.107512.

[ref13] Gan C.; Cao W. H.; Liu K. Z.; Wu M.; Wang F. W.; Zhang S. B. A new hybrid bat algorithm and its application to the ROP optimization in drilling processes. IEEE Trans. Ind. Inf. 2020, 16, 7338–7348. 10.1109/TII.2019.2943165.

[ref14] Li W. Y. Fractal theory and modified rate of penetration equation to predict drillability of gypsum interbedded rocks. West China Explor. Eng. 2022, 34, 52–58.

[ref15] Chen X. Y.; Gao D. L.; Guo B. Y.; Feng Y. C. Real-time optimization of drilling parameters based on mechanical specific energy for rotating drilling with positive discharge of pump motor in the hard formation. J. Nat. Gas Sci. Eng. 2016, 35, 686–694. 10.1016/j.jngse.2016.09.019.

[ref16] Chen X. H. Application of drilling efficiency optimization using mechanical specific energy theory in daniudi gas field. Drill. Prod. Tech. 2017, 40, 28–33.

[ref17] Cui M.; Li J. J.; Ji G. D.; Chen Y. H. Optimize Method of Drilling Parameter of Compound Drilling Based on Mechanical Specific Energy Theory. Pet. Drill. Tech. 2014, 42, 66–70. 10.3969/j.issn.1001-0890.2014.01.013.

[ref18] Yang L.; Liu T. Y.; Ren W. J.; Sun W. F. Fuzzy Neural Network for Studying Coupling between Drilling Parameters. ACS Omega 2021, 6, 24351–24361. 10.1021/acsomega.1c02107. 34604618 PMC8482398

[ref19] Ahmed A.; Ali A.; Abdulraheem A. New Artificial Neural Networks Model for Predicting Rate of Penetration in Deep Shale Formation. Sustainability 2019, 11, 6527 10.3390/su11226527.

[ref20] Kor K.; Altun G. Is Support Vector Regression method suitable for predicting rate of penetration?. J. Pet. Sci. Eng. 2020, 194, 107542 10.1016/j.petrol.2020.107542.

[ref21] Ashrafi S. B.; Anemangely M.; Sabah M.; Ameri M. J. Application of hybrid artificial neural networks for predicting rate of penetration (ROP), A case study from Marun oil field. J. Pet. Sci. Eng. 2019, 175, 604–623. 10.1016/j.petrol.2018.12.013.

[ref22] Tan Y.; Zheng S. Q. Recent advances in fireworks algorithm. CAAI Trans. Intell. Syst. 2014, 9, 515–528.

[ref23] Wang S. D.; Zhao T. Y.; Pang S. C. Task scheduling algorithm based on improved firework algorithm in fog computing. IEEE Access 2020, 8, 32385–32394. 10.1109/ACCESS.2020.2973758.

